# Mobile Phone–Based Intervention Among Adolescents Living With Perinatally Acquired HIV Transitioning from Pediatric to Adult Care: Protocol for the Interactive Transition Support for Adolescents Living With HIV using Social Media (InTSHA) Study

**DOI:** 10.2196/35455

**Published:** 2022-01-21

**Authors:** Brian C Zanoni, Moherndran Archary, Thobekile Sibaya, Madeleine Goldstein, Scarlett Bergam, David Denton, Vincente Cordero, Cynthia Peng, Christina Psaros, Vincent C Marconi, Jessica E Haberer

**Affiliations:** 1 School of Medicine Emory University Atlanta, GA United States; 2 Children's Healthcare of Atlanta Atlanta, GA United States; 3 Rollins School of Public Health Emory University Atlanta, GA United States; 4 Nelson R. Mandela School of Medicine University of KwaZulu-Natal Durban South Africa; 5 King Edward VIII Hospital Durban South Africa; 6 Massachusetts General Hospital Boston, MA United States; 7 Harvard Medical School Harvard University Boston, MA United States

**Keywords:** adolescent, mHealth, South Africa, HIV, Social media, InTSHA, protocol, transition support, support, WhatsApp, caregiver, health care provider

## Abstract

**Background:**

Adolescents living with perinatally acquired HIV often have poor retention in care and viral suppression during the transition from pediatric to adult-based care.

**Objective:**

The aim of this study is to evaluate a mobile phone–based intervention, Interactive Transition Support for Adolescents Living With HIV using Social Media (InTSHA), among adolescents living with perinatally acquired HIV as they transition from pediatric to adult care in South Africa.

**Methods:**

InTSHA uses encrypted, closed group chats delivered via WhatsApp (Meta Platforms Inc) to develop peer support and improve communication between adolescents, their caregivers, and health care providers. The intervention is based on formative work with adolescents, caregivers, and health care providers and builds on several existing adolescent support programs as well as the Social-ecological Model of Adolescent and Young Adult Readiness for Transition (SMART). The final InTSHA intervention involves 10 modules conducted weekly through moderated WhatsApp group chats with adolescents and separately with their caregivers. We will randomly assign 80 South African adolescents living with perinatally acquired HIV who are aware of their HIV status and aged between 15 and 19 years to receive either the intervention (n=40) or standard of care (n=40).

**Results:**

We will measure acceptability of the intervention as the primary outcome and evaluate feasibility and preliminary effectiveness for retention in care and viral suppression after completion of the intervention and at least 6 months after randomization. In addition, we will measure secondary outcomes evaluating the impact of the InTSHA intervention on peer support, self-esteem, depression, stigma, sexual education, connection to health care providers, and transition readiness. Enrollment began on April 15, 2021. As of December 31, 2021 a total of 78 out of expected 80 participants have been enrolled.

**Conclusions:**

If successful, the intervention will be evaluated in a fully powered randomized controlled trial with a larger number of adolescents from urban and rural populations to further evaluate the generalizability of InTSHA.

**Trial Registration:**

ClinicalTrials.gov NCT03624413; https://clinicaltrials.gov/ct2/show/NCT03624413

**International Registered Report Identifier (IRRID):**

DERR1-10.2196/35455

## Introduction

South Africa has the largest antiretroviral therapy (ART) program along with highest burden of adolescents living with HIV (ALHIV) in the world [1]. Survivors of perinatally acquired HIV are now reaching adolescence and beyond, yet the majority of adolescents are poorly prepared for the transition from pediatric to adult care services [2,3]. An estimated 320,000 adolescents with perinatally acquired HIV will transfer from pediatric-based or adolescent-based clinics to adult services in the next 10 years in South Africa [4]. Currently, adolescents living with perinatally acquired HIV transition to adult care at variable ages and developmental stages, without necessary preparation or support throughout the process [5]. Guidelines for transitioning adolescents to adult care are often based on age and are not tailored to the adolescents’ developmental levels or needs. The transition from pediatric to adult services is also a challenging time during which clinical outcomes commonly suffer [2,6,7]. In South Africa, adolescents transitioning to adult care have shown lower viral suppression rates than those remaining in pediatric care [2]. Effective interventions are clearly needed to improve clinical outcomes in this highly vulnerable population, particularly in older adolescents transferring clinical care.

Medical care during adolescence is typically complicated by increased risk-taking behavior as well as decreased caregiver involvement, which occur during a time of rapid physical, emotional, and cognitive development [8-11]. When adolescents transition to adult care, they often do not receive the coordinated services that they received under pediatric care [12]. Qualitative studies with adolescents and clinicians from sub-Saharan Africa suggest that peer support, collaboration with health care providers, and communication between adult and pediatric providers might improve the transition to adult services [5,13,14]. The Social-ecological Model of Adolescent and Young Adult Readiness for Transition (SMART) highlights modifiable targets of intervention, such as knowledge, skills/self-efficacy, relationships, and social support, that can be addressed to improve the transition of care [15,16]. It further lays out the roles of 3 key stakeholders (adolescents, caregivers, and health care providers) and their interconnected relationship in influencing a successful transition to adult care. This model thus offers a potential structure with which to design a social support intervention [15-17].

The use of social media and access to mobile phones among adolescents in South Africa is growing rapidly and offers an excellent opportunity to deliver a social support intervention [18]. Social media is defined as Internet-based applications that allow the creation and exchange of user-generated content; examples include WhatsApp (Meta Platforms Inc) and Facebook (Meta Platforms Inc) [19]. Social media has been shown to support change across several modifiable factors in SMART, such as relationships, social support, and knowledge, in other contexts. A meta-analysis found that social support was the most common reason for patients to use social media for health purposes [18]. Social media has also been used to improve the relationship between caregivers and patients when switching caregivers, a major barrier to transition for ALHIV in South Africa [20,21]. Although results vary in different settings, another meta-analysis showed overall improved adherence to ART and viral suppression among adults living with HIV using social media–based health services technology [22]. Social media may also be able to address additional modifiable factors in SMART, such as self-efficacy and goal development, which could ultimately improve virologic suppression and retention in care during the transition to adult services [23].

In this paper, we describe the research protocol for the pilot clinical trial involving Interactive Transition Support for Adolescents Living With HIV using Social Media (InTSHA).

## Methods

### InTSHA Intervention

We created a preliminary version of InTSHA as indicated in Table 1, using a participant-centered approach and based on formative interviews with adolescents, caregivers, and health care providers, which will be published separately. In addition, we used SMART [15] to guide intervention development. We also used the in-person adolescent support group discussion material developed by the US Agency for International Development, as part of the Right to Care Flipster tool [24], as a starting point for the development of the 10 skill-building modules. We used Got Transition’s Six Core Elements of Health Care Transition [25] to structure the delivery of the clinic’s transition policy through WhatsApp messaging. Got Transition also calls for tracking and monitoring of adolescents’ progress that we organized through 2-way messaging between adolescents and care providers. To delve into transition readiness and planning itself, we used SMART, focusing on modifiable factors that can be addressed through content delivery, facilitated discussions, web-based meet ups and consultation with the health care team. InTSHA then concludes with WhatsApp discussions between adult and pediatric clinicians through the final 2 elements of Got Transition, transfer of care, and transfer completion.

**Table 1:** Content and design of the initial Interactive Transition Support for Adolescents Living With HIV using Social Media (InTSHA) intervention.

Got Transition elements	Modifiable SMART^a^ factors	Intervention component
Transition policy	N/A^b^	Direct content delivery
Transition tracking/monitoring	N/A	2-way messaging between adolescents and care providers
**Transition readiness**		
	Knowledge, skills	Content delivery
	Maturity, goals, beliefs, self-efficacy	Facilitated discussions
**Transition planning**		
	Relationships, social support	Web-based meet ups/discussions
	Clinical support	Health care team consultation
Transfer of care	N/A	Discussions between adult and pediatric clinicians
Transfer completion	N/A	Discussions between adult and pediatric clinicians

^a^SMART: Social-ecological Model of Adolescent and Young Adult Readiness for Transition.

^b^N/A: not applicable.

Participants randomized to the intervention group will receive the InTSHA intervention. The intervention consists of 10 modules which will be delivered weekly by group chat through a closed, encrypted, and password-protected WhatsApp chat group based on preferences determined in formative research. Modules contain topics of interest voiced by formative qualitative interviews and include the following: (1) web-based security, (2) HIV disclosure, (3) drug and substance abuse, (4) sexual and reproductive health, (5) gender roles and sexuality, (6) stigma, (7) HIV knowledge and health care navigation, (8) ART adherence and HIV resistance, (9) healthy relationships, and (10) career planning and future goals. Closed chat groups will consist of mixed genders and up to 10 ALHIV and will be facilitated by a research coordinator. Modules will take place during weekly scheduled sessions with 1 additional brief check-in session per week. Adolescents will have access to the chat group outside of scheduled discussion times to check in with group members, review the content of the sessions, or comment or ask additional questions. Adolescents will also have the opportunity to ask health-related questions to health care providers at the clinic via WhatsApp. Questions are facilitated by the research coordinator to prevent negative messaging and to minimize potential social harm. All discussions are monitored by clinical doctors trained in pediatric HIV for accuracy and clarity. To ensure access to mobile data during scheduled chats, 1 gigabyte of data will be loaded onto each participant’s phone prior to scheduled chat discussions.

Caregivers of adolescent participants will participate in separate closed, encrypted, and password-protected WhatsApp chat groups with weekly topics mirroring the adolescent topics to facilitate discussions between adolescents and their caregivers.

### Statement of Ethics

The Biomedical Research Ethics Committee of the University of KwaZulu-Natal, KwaZulu-Natal Department of Health, Mass General Brigham (formerly Partners HealthCare) Research Ethics Board, and Emory University Institutional Review Board approved this protocol.

### Clinical Trial Research Protocol

We will evaluate the InTSHA intervention in a pilot randomized controlled clinical trial among ALHIV transitioning to adult care. We will measure acceptability, feasibility, and preliminary efficacy of the InTSHA intervention by measuring viral suppression 6 months after transitioning to the adult clinic among the intervention (n=40) and control (n=40) groups.

### Participant Selection, Enrollment, and Randomization

Adolescents living with perinatally acquired HIV between the ages of 15 and 19 years from KwaMashu Poly Clinic, KwaMashu, South Africa, and Mahatma Gandhi Memorial Hospital, Durban, South Africa, will be offered enrollment during their routine outpatient appointments if they have been on ART for at least 6 months and are fully aware of their HIV status. Participants will be randomized (1:1) to receive the InTSHA intervention delivered via WhatsApp closed chat groups or standard of care. Randomization will be performed using sealed envelopes containing study assignments. The research team will be blinded to the contents of the envelopes, which will be created using block randomization by a computer-generated random number sequence, then filled and sealed by non–research staff.

### Participant Consent

All participants under the age of 18 years will be required to provide written assent to participation in this study. Written informed consent from caregivers will be obtained for adolescents less than 18 years old. Adolescents 18 years or older will provide their own written consent. Assent and/or consent forms will be offered in both English and isiZulu.

### Standard of Care

Participants in the standard of care arm will continue usual care at KwaMashu Poly Clinic or Mahatma Gandhi Memorial Hospital. Adolescents typically transition to adult care after the age of 15 years if they are aware of their HIV status and taking a fixed-dose combination ART. In the adult clinic, adolescents are seen together with adults in a general clinic that also attends to patients with other chronic illnesses. Young adults with perinatally acquired HIV and behaviorally acquired HIV are seen in the same clinic. Patients are seen by a health care provider every 3 months and collect medication monthly at an on-site pharmacy.

### Procedures

During visit 1, we will collect baseline demographic data, complete baseline questionnaires, and perform viral load assessments for all participants. The second research visit will take place either 6 months after randomization or after completion of all 10 modules, whichever comes last; although the modules are designed to be completed over 10 consecutive weeks, we anticipate additional time may be needed to accrue an adequate number of participants into the group intervention and alternate scheduling may be needed to accommodate school demands, holidays, and other events. The second research visit will evaluate acceptability and feasibility and perform exit focus group discussions with all participants in the intervention arm. In addition, all subjects will complete follow-up questionnaires and have a viral load assessment.

We will collect demographic data on all adolescents using chart review and will include age, sex, age at diagnosis, history of opportunistic infections, length of time on ART, ART regimen, last viral load, clinic visits, and pharmacy refill information. Data will be collected from a combination of electronic records and paper charts. Additional data on viral status and location of care services will be obtained through an existing patient tracker program.

### Ethical Considerations

To address participant concerns voiced during formative work, we have included multiple safeguards. To protect privacy and prevent accidental disclosure of HIV status if others use or share phones, we will ensure that all phones are password-protected. The individual chat discussions will be encrypted and password-protected within WhatsApp. In addition, all participants will choose a pseudonym and actual names will not be visible within the chats. If an adolescent communicates physical or emotional distress, including suicidality, the facilitator and health care providers have established referral networks and collated resources to provide the adolescent. To ensure accuracy of information provided within the chats, the content will be reviewed by the research team and supervising physician.

## Results

### Primary and Secondary Outcomes

We will determine the primary outcomes of acceptability (acceptability of implementation measure) and feasibility (enrollment, participation, and feasibility of implementation measure) [26]. We will also measure the secondary outcomes of retention in care (pharmacy refill and clinic attendance in the last 6 months) and viral suppression (<400 copies/mL), comparing pretransition with 6 months after randomization. In addition, we will explore other factors based on modifiable variables in SMART including (as indicated in Table 2) the following: adolescent peer support (using the Child and Adolescent Social Support Scale) [27], self-esteem (using the Rosenberg Self-Esteem Scale) [28], depression (using the Patient Health Questionnaire-9 [PHQ-9]) [29], stigma (using the Internalized AIDS-Related Stigma Scale) [30], connection to clinic (using the Working Alliance Inventory) [31], and transition readiness (using the HIV Adolescent Readiness for Transition Scale) [32].

**Table 2:** Factors associated with SMART^a^ and measures to evaluate the impact of the InTSHA^b^ intervention on each.

Factors associated with SMART	Measure
Peer support	Child and Adolescent Social Support Scale
Self-esteem	Rosenberg Self-Esteem Scale
Depression	PHQ-9^c^
Stigma	Internalized AIDS-Related Stigma Scale
Connection to clinic	Working Alliance Inventory
Transition readiness	HIV Adolescent Readiness for Transition Scale

^a^SMART: Social-ecological Model of Adolescent and Young Adult Readiness for Transition.

^b^InTSHA: Interactive Transition Support for Adolescents Living With HIV using Social Media.

^c^PHQ-9: Patient Health Questionnaire-9.

### Analysis

We will summarize the acceptability and feasibility data in the intervention group using standard summary statistics (eg, counts or percentages, median and interquartile range of continuous measures). We will estimate the difference between the intervention group and the control group for social support, connection to clinic, self-esteem, stigma, retention in care, and virologic suppression 6 months after randomization. The standard deviation for these differences is expected to be on the order of 8%. We will use logistic regression to explore combinations of variables potentially predictive of retention in care and viral suppression. The number of variables analyzed will depend on the number of individuals with each outcome.

### Power Analysis for Primary Outcome

This study is powered on the primary outcome of acceptability. With 40 participants in the intervention arm, if the reported acceptability rate is 90%, we will be able to rule out that acceptability was less than 70% with 90% power (α=0.05, one-sided) using a Fisher exact test.

### Timeline

Enrollment began on April 15, 2021. As of December 31, 2021 a total of 78 out of expected 80 participants have been enrolled.

## Discussion

Increasing evidence indicates poor clinical retention in care and viral suppression among many adolescents living with perinatally acquired HIV who are transitioning from pediatric to adult care [2,33]. Despite the uptake of social media and mHealth interventions to address gaps in many aspects of the HIV continuum of care in low-income and middle-income income countries (LMIC), there are no interventions addressing the transition to adult care (unpublished study by Goldstein et al). InTSHA is a social media intervention using existing technology that aims to fill this gap. Using a participant-centered design among the 3 key stakeholders—adolescents, caregivers, and health care providers—we created an intervention based on SMART, Got Transition, and the Right to Care Flipster tool by addressing modifiable factors, such as knowledge, skills/self-efficacy, relationships, and social support. To our knowledge, only one other ongoing study is using mHealth to support the transition from pediatric to adult care for adolescents and young adults living with HIV. Specifically, Tanner et al [34] are evaluating a novel smartphone app, iTransition, based on social cognitive theory to support ALHIV in the United States. However, the development, evaluation, and implementation of effective and accessible mHealth interventions are needed to improve health care transition outcomes for adolescents living with perinatally acquired HIV in LMIC, where the majority of youth living with perinatally acquired HIV reside.

Mobile phone ownership and use for health interventions has increased worldwide in recent years [18]. This approach is particularly promising for adolescents who are avid adopters of technology, although few mHealth interventions have shown to be effective at engaging adolescents. Although mHealth technology has the ability to address many barriers to transitional care and improve communication, several practical barriers remain. Socioeconomic pressure from the cost of mobile phones and data plans can be prohibitive for younger adolescents, particularly in LMIC, to engage in mHealth. The practice of sharing phones can reduce associated costs, but privacy and confidentiality may be compromised [35]. In addition, inferior mobile network coverage and limited technological literacy in LMIC can limit the effectiveness of mHealth interventions. Despite these potential barriers, InTSHA is likely to benefit adolescents by providing social support and connections to clinical staff as they transition to adult care. Therefore, studies evaluating the real-world uptake, effectiveness, and cost of mHealth interventions are required if they are going to be utilized and sustained for meaningful impact [36].

InTSHA is a theory-based, participant-centered intervention to support the transition from pediatric care to adult care that will be rigorously evaluated for acceptability, feasibility, and preliminary effectiveness using real-world conditions. If successful, this intervention will be formally tested in a fully powered randomized controlled trial including multiple populations of adolescents for increased generalizability of effect. Such work has great potential to improve the poor outcomes associated with transitioning to adult care for thousands of adolescents living with HIV globally.

## Appendix

Multimedia Appendix 1 Peer-review report by the Center for Scientific Review Special Emphasis Panel - Member Conflict: AIDS and AIDS Related Research (National Institutes of Health, USA).

## Abbreviations

ALHIV: adolescents living with HIV
ART: antiretroviral therapy
InTSHA: Interactive Transition Support for Adolescents Living With HIV using Social Media
LMIC: low-income and middle-income income countries
PHQ-9: Patient Health Questionnaire-9
SMART: Social-ecological Model of Adolescent and Young Adult Readiness for Transition
