# Supplementary material for: Mobile Phone–Based Intervention Among Adolescents Living With Perinatally Acquired HIV Transitioning from Pediatric to Adult Care: Protocol for the Interactive Transition Support for Adolescents Living With HIV using Social Media (InTSHA) Study
Source: JMIR Res Protoc. 2022 Jan 21;11(1):e35455. doi: 10.2196/35455 (PMC8817214; doi:10.2196/35455)
Supplement: Multimedia Appendix 1 [file resprot_v11i1e35455_app1.pdf]

SUMMARY STATEMENT
(Privileged Communication)

PROGRAM CONTACT:
Susannah Allison
240-627-3861
allisonsu@mail.nih.gov

Release Date: **12/15/2017**
Revised Date:

Application Number: **1 K23 MH114771-01A1**

Principal Investigator

ZANONI, BRIAN C.

Applicant Organization: MASSACHUSETTS GENERAL HOSPITAL

Review Group:	ZRG1 AARR-F (02)<br>Center for Scientific Review Special Emphasis Panel<br>Member Conflict: AIDS and AIDS Related Research<br>AIDS
Meeting Date:	11/22/2017
Council:	JAN 2018
Requested Start:	04/01/2018
RFA/PA:	PA16-198
PCC:	9A-ASPA

Project Title:	InTSHA: Interactive Transition Support for HIV-infected Adolescents Using Social Media
SRG Action:	Impact Score:20
Next Steps:	Visit https://grants.nih.gov/grants/next_steps.htm
Human Subjects:	30-Human subjects involved - Certified, no SRG concerns
Animal Subjects:	10-No live vertebrate animals involved for competing appl.
Gender:	1A-Both genders, scientifically acceptable
Minority:	5A-Only foreign subjects, scientifically acceptable
Children:	1A-Both Children and Adults, scientifically acceptable<br>Clinical Research - not NIH-defined Phase III Trial

Project Year	Direct Costs Requested	Estimated Total Cost
1	182,973	197,611
2	182,797	197,421
3	182,565	197,170
4	182,622	197,232
TOTAL	730,957	789,434

ADMINISTRATIVE BUDGET NOTE: The budget shown is the requested budget and has not been adjusted to reflect any recommendations made by reviewers. If an award is planned, the costs will be calculated by Institute grants management staff based on the recommendations outlined below in the COMMITTEE BUDGET RECOMMENDATIONS section.

1K23MH114771-01A1 Zanoni, Brian

RESUME AND SUMMARY OF DISCUSSION: This accomplished candidate seeks to become an independent investigator with an interest in the adaptation and evaluation of interventions to improve transition care for adolescents living with HIV in South Africa. The candidate, is very productive (8 first author publications) and has conducted research in Botswana and South Africa that is relevant to his current career goals. He will acquire additional training in qualitative research methods for behavioral intervention design, adaptation of social media technologies, and clinical trials for behavioral interventions. He has presented a training plan that consists of formal coursework, conferences, directed readings, and mentoring. This plan appropriately covers areas in which the candidate requires instruction; it is well balanced. For his research plan, the candidate will develop and evaluate a social media intervention based on the Social-ecological Model of Adolescent and Young Adult Readiness to Transition (SMART). This model will include an assessment of knowledge, beliefs, and motivation about transition from pediatric to adult care. He will then adapt an existing social media tool (e.g., WhatsApp) with input from adolescents, caregivers, and healthcare providers, and will test the acceptability and feasibility of this intervention among adolescents living with HIV who are transitioning to adult care in South Africa. The focus of this research is timely and very important given that South Africa has the highest number of perinatally HIV infected children in the world. Moreover, as these children transition from adolescence, when they are integrated in HIV treatment, to adulthood, there is disengagement in the care cascade, leading to low viral suppression, morbidity, and high mortality. To assist him with his objectives, the candidate has retained an outstanding group of mentors with the relevant expertise to guide him in this endeavor. It is evident they are committed to his success in light of the very glowing letters of support they have written on his behalf. This resubmission was very responsive to prior critiques; the training plan and research aims are better aligned with one another; he has also reduced his mentoring team by four mentors and added two whose expertise is relevant to the candidate’s training plan and research goals; the application is vastly improved. There were weaknesses raised about the application; however, these were considered minor and only slightly dampened enthusiasm. There were initially reservations on the part of some reviewers about the need for an already well qualified candidate for additional training; however, the candidate made a compelling case for training in qualitative analysis, evaluation of social media, or clinical trials for behavioral interventions, skills he felt he lacked. The committee was convinced, and therefore, extends very high support for this meritorious candidate who will without a doubt become an independent investigator and contribute to the field.

DESCRIPTION (provided by applicant): South Africa has the highest burden of adolescents living with HIV in the world. Clinical outcomes are poor for adolescents during the transition period. Social media is popular among youth and has potential to improve the modifiable factors in the Social-ecological Model of Adolescent and Young Adult Readiness to Transition (SMART) model such as knowledge, self-efficacy, beliefs, maturity, goals, relationships, peer and social support. The SMART model highlights these modifiable targets of intervention and presents an ideal basis for a behavioral intervention using social media to improve transition care. Innovation: This study will iteratively develop a social media behavioral intervention for adolescents living with HIV and transitioning to adult care in South Africa. This proposal is the first intervention, to our knowledge, designed to address transition outcomes (i.e., retention in care and viral suppression) for HIV-infected adolescents in sub-Saharan Africa where 90% of the pediatric HIV-infections exist. Hypothesis: I hypothesize that an iteratively developed social media based behavioral intervention will be acceptable and feasible for adolescents living with HIV and transitioning to adult care. Approach: I will use in-depth qualitative interviews to determine how social media can overcome barriers and enhance facilitators of successful transition to adult care for adolescents living with HIV in South Africa (Aim 1). I will then iteratively adapt the social media based behavioral intervention, Interactive Transition Support for HIV-infected Adolescents (InTSHA; meaning youth in Zulu), using focus groups with adolescents, their caregivers, and healthcare providers (Aim 2). I will then conduct a pilot clinical trial of the intervention (Aim 3) to determine its

acceptability and feasibility. Training Aims: I will undergo didactic study with coursework from the Harvard TH Chan School of Public Health, Harvard Kennedy School, Massachusetts Institute of Technology, and the NIH Office of Social Science and Behavioral Research in: Qualitative research methods for behavioral intervention design, social media use for behavioral interventions, and clinical trials of behavioral interventions. In addition, I will have didactic training with my mentors, attend and present at regular research seminars at Massachusetts General Hospital, conduct formative readings, and participate in the Adolescent Clinical Trials Network (ATN) social media study, iTech. Investigator: Brian Zanoni trained in Internal Medicine/Pediatrics with specialization in Infectious Disease, has extensive clinical experience in adolescent HIV care in South Africa, a strong research foundation, and ample mentorship. Mentors: Jessica Haberer is an expert in global health technology for HIV and serves as Dr. Zanoni's primary mentor. Christina Psaros is a behavioral scientist and clinical psychologist with expertise in using qualitative research methods for psychosocial aspects of HIV care. Richard Holden is an expert in mobile health technology and behavioral interventions. Kenneth Mayer is an internationally recognized leader in HIV interventions, clinical trials and site principal investigator for the ATN.

PUBLIC HEALTH RELEVANCE: South Africa has the highest number of perinatally HIV-infected children in the world. With antiretroviral therapy, these children are surviving into adulthood and will need to transition to adult care to achieve long-term successful outcomes. Adolescents living with HIV and transitioning to adult care have shown high rates of mortality, virologic failure, and loss to follow up. The proposed research will develop and evaluate a social media behavioral intervention based on the Social-ecological Model of Adolescent and Young Adult Readiness to Transition (SMART).

CRITIQUE 1

Candidate: 1
Career Development Plan/Career Goals /Plan to Provide Mentoring: 2
Research Plan: 3
Mentor(s), Co-Mentor(s), Consultant(s), Collaborator(s): 1
Environment Commitment to the Candidate: 1

Overall Impact: This revised application is submitted by a talented and dedicated candidate who hopes to advance a social media intervention to aid transition of adolescents to adult HIV care. The proposal envisions a qualitative aim to obtain input from adolescents, caregivers and care providers, a second aim to refine a social media tool focused on improving transition using a SMART approach and adapting principles/components from Got Transition, and finally a pilot RCT of the the social media tool inTSHA. Strengths of the application include the very committed and productive candidate – trained in Med/Peds with extensive experience providing pediatric and adolescent HIV care in Botswana and South Africa, motivated by specific pragmatic clinical needs of transitioning children. He has been highly productive with 8 first author publications (many focused on ped/adolescent HIV) and several training and small grants (Including an R21 for which he is PI). The mentoring team is superb – with directly relevant expertise and a clear commitment of mentoring time – and complementary expertise. The research aims address a high priority area and advance an idea that could be expanded in an R01 to identify a relevant tool for use in SA or other settings. The qualitative and social media aims dovetail well with the training plan in these domains. The training plan is well outlined and includes didactic and mentored training. The environment is outstanding. Weaknesses of the application include the perhaps over-ambitious training plan. The research involves development of a social media tool that is complex and involves multiple components and may be difficult to dependably replicate or generalize. The enrollment period and follow-up may make It difficult to have a homogeneous coverage of the intervention. Other minor analytic challenges and a lack of pilot data on influence on transition were

minor weaknesses. Overall, an excellent application, responsive to the prior critiques with minor weaknesses.

1. Candidate:

Strengths

- The candidate is exceptionally well qualified – trained in Internal Medicine and Pediatrics at Baylor with quite extensive global health experience in Botswana and South Africa – his passion for the topic stems from a touching personal encounter with a child with HIV who had challenges as she became an adolescent.
- Dr. Zanoni also obtained an MPH in Clinical Effectiveness from Harvard and completed his Infectious Diseases Fellowship at MGH and clinical HIV training (1 year). He sought out mentorship from Dr. Haberer specifically to provide guidance on research to improve adolescent ART adherence.
- Dr. Zanoni founded a Teen Clinic in South Africa with a focus on transition which is directly relevant to his K23 research focus.
- Candidate has 8 first author publications in excellent journals – including observational analyses of pediatric and adolescent cohorts, longitudinal studies and systematic reviews – he is clearly a 'finisher' and has demonstrated success in accessing diverse funding (T32, KL2, CFAR, R21) – all clearly pointing his high likelihood of success as a research scientist.

Weaknesses

- Prior critiques noted that in some ways he has met many of the milestones 'post-K' and seems somewhat overqualified for a K23 given his training, high productivity and grant success. However, this is a minor weakness and does not detract from his exceptional qualifications as a candidate. His rationale for the K is that additional qualitative training and focus on transition will provide him the best foundation for long-term career.

2. Career Development Plan/Career Goals & Objectives/Plan to Provide Mentoring:

Strengths

- Candidate has several training aims. The first aim is to gain skills in qualitative research – with didactic coursework and twice monthly hands-on training with Dr. Psaros – in depth interviews/focus group discussions, coding etc., which directly aligns with his proposed research Aim 1. The skills proposed will complement candidate's quantitative background.
- Directed readings, mhealth working groups, and interactions with MIT-affiliated SANA group (on mhealth) are proposed to complement Aim 2. Interactions with an ongoing ATN RCT of mhealth intervention are proposed to complement Aim 3. Thus, the training aims align well with the research aims.
- Weekly meetings are proposed with the primary mentor (Haberer) – similar to current mentoring which has clearly been effective to date. Twice monthly meetings are proposed with Dr. Psaros for qualitative research, and for the mhealth and adolescent HIV/RCT mentoring with Drs. Holden and Mayer. The frequency of meetings seems appropriate and likely to be effective.

Weaknesses

- The reach of disciplines and training remains ambitious – spanning qualitative, behavioral, and clinical trial training. Whether the candidate will continue in any of the qualitative, behavioral domains as a focus or simply use the training to establish conversancy and collaborations is unclear. Ultimately, as a clinical researcher, the aim to implement an effective intervention is

clearly preeminent – and understanding mechanism and designing appropriate interventions are a part of this. The combination of training domains is reasonable but still seems ambitious.

3. Research Plan:

Strengths

- The study proposes a fairly conventional sequence of development – including qualitative in depth interviews with adolescents, caregivers, and health care workers regarding transition followed by mhealth application development (using behavioral underpinning) and a pilot RCT evaluating a whatsapp intervention using the developed tool. The combination of aims is feasible during a 5-year period and could be scaled for a larger RCT R01.
- Given the large population of transitioning HIV infected adolescents in SA, this strategy could have significant public health benefits.
- The Mahatma Gandhi hospital has a large cohort of HIV infected children and adolescents and the candidate envisions obtaining data from children not yet transitioned, successfully transitioned, and lost – the candidate has previously tracked 'lost' adolescents which will provide particularly relevant data.
- The SMART model and the domains to be incorporated in the new social media tool are a reasonable approach. The InTSHA does not require development of a new app/tool but will use an existing tool and incorporate specific content/discussions focused on optimized transition.
- Clearly articulated milestones/deliverables are envisioned with target analyses/manuscripts and journals specified.

Weaknesses

- The candidate has outlined a fairly comprehensive list of pitfalls and limitations in the application – small RCT, limited duration of follow-up, underpowered for hard outcomes, all of which are reasonable limitations within the scope of the K23/pilot.
- The intervention itself depends on a variety of formats – educational content, discussion groups, input from care providers, and peer groups – fidelity and replicability will be challenging given the multifactorial nature of the intervention.
- The complexity of the intervention may limit generalizability.
- The peer component clusters groups in the intervention arm which introduces some analytic challenges.
- The outcomes of retention and viral suppression, while interesting are only indirectly related to the focus on transition – transitional readiness is included and may be a useful marker but largely depends on the age group of the enrollees
- A 15 month enrollment period is envisioned with unclear 'dose'/duration consistency of the intervention given the long enrollment period.
- It is possible that clinicians and peers may contaminate those in the 'control' group with diffusion of the concepts covered in the curriculum in inTSHA.

4. Mentor(s), Co Mentor(s), Consultant(s), Collaborator(s):

Strengths

- Mentoring team includes complementary expertise – Dr. Haberer provides expertise on adherence, Dr. Psaros on behavioral/qualitative research, Dr. Holden on mobile health, and Dr.

Mayer on clinical trials/adolescent HIV. The team has been adapted from the prior application – streamlining by decreasing 4 members and trying to align better with his research aims.

- Dr. Haberer has an outstanding track record for both her own scientific portfolio as well as for mentoring – having received a K24 and a Harvard Medical School Young Mentor Award. She has served as the candidate's KL2 mentor and has successfully mentored other mentees to publications and to independent NIH funding.
- The candidate has also proposed to convene a Scientific Advisory Board with Dr. Mellins (adolescent behavioral health interventions), Drs. Mossa and Archary (SA adult/pediatric HIV), Dr. Parker (biostat/clinical trials). Advisory Board will be convened annually.

Weaknesses

- None noted.

5. Environment and Institutional Commitment to the Candidate:

Strengths

- The environment both at Harvard and in South Africa are excellent with appropriate infrastructure, collaborative history, and resources.

Weaknesses

- None

Protections for Human Subjects:

Acceptable Risks and Adequate Protections

- has included discussion of appropriate considerations - particularly loss of confidentiality

Data and Safety Monitoring Plan (Applicable for Clinical Trials Only):

Not Applicable (No Clinical Trials)

Inclusion of Women, Minorities and Children:

- Sex/Gender: Distribution justified scientifically
- Race/Ethnicity: Distribution justified scientifically
- For NIH-Defined Phase III trials, Plans for valid design and analysis: Not applicable
- Inclusion/Exclusion of Children under 18: Including ages < 18; justified scientifically
- To be conducted in SA - a region with the highest burden of pediatirc HIV globally - includes female/male equally and children

Vertebrate Animals:

Not Applicable (No Vertebrate Animals)

Biohazards:

Not Applicable (No Biohazards)

Reubmission:

- Applicant has been responsive to prior critiques

Training in the Responsible Conduct of Research:
Acceptable
Comments on Format (Required):

- included prior training CITI, GCP certification

Comments on Subject Matter (Required):

- Broad range of issues is listed

Comments on Faculty Participation (Required; not applicable for mid- and senior-career awards):

- lectures and group seminars are included

Comments on Duration (Required):

- specified per year (.>20-30 hours)

Comments on Frequency (Required):

- lectures, group discussions, faculty involvement anticipated each year

Resource Sharing Plans:
Acceptable

Budget and Period of Support:
Recommend as Requested

CRITIQUE 2

Candidate: 2
Career Development Plan/Career Goals /Plan to Provide Mentoring: 3
Research Plan: 3
Mentor(s), Co-Mentor(s), Consultant(s), Collaborator(s): 2
Environment Commitment to the Candidate: 1

Overall Impact: In this revised application, the candidate proposes a career development plan to strengthen his knowledge of qualitative methods for behavioral intervention design, use of social media platforms for promoting adolescent behavior change and clinical trials for testing behavioral interventions. This is a promising candidate who is well positioned to achieve research independence. The candidate thoughtfully addressed many of the key points raised in the prior critique. The candidate adequately described his long term career goal and how the proposed training and research in this K-23 application will help him to address the skills deficits he currently has to achieve his long term career goals. The size of the mentoring team was reduced to 4 mentors, each experienced in each of the training areas. The proposed research is scientifically rigorous and maps on well to the training objectives. The mentors' letters are strong and outline their contributions to the project. Institutional support is also high and clearly delineates the candidate's level of effort on the K. The domestic as well as the South African research environment are also excellent. The only concern identified in this

review is that the training plan articulated deliverables (all of which are publications), but specific milestones that could be used to assess progress are not described

1. Candidate:

Strengths

- The candidate is a promising young investigator with a good publication record (8 first author papers and 9 others as co-author). His publications are in high impact journals and are of high quality
- He has strong clinical experience working with adolescents in resource poor setting. Of particular relevance to the current application is the 5 years he spent on the ground working in South Africa delivering services to youth living with HIV. As a result of the needs he encountered, he founded a clinic to serve the needs of youth living with HIV
- He has been a part of two T32 programs and has received an internal career development grant (KL2)
- The candidate statement and the mentor letters attest to the latter's commitment to the former's dedication to become an independent researcher and pursue a youth-focused HIV research career and the latter's commitment to the candidate's success.
- The candidate's bio and the mentors' strong letters convincingly argue that the candidate has strong potential to achieve research independence
- While the candidate's prior training does not provide a strong foundation for the work proposed in this application, it supports the candidate's need to secure additional training in qualitative methods, behavioral interventions and social media applications via a K-award to achieve his career objectives.

Weaknesses

- The candidate's prior training has been in HIV medical management, Infectious disease, tropical medicine, not intervention development or behavior change, which provide a limited foundation for the research proposed in the application

2. Career Development Plan/Career Goals & Objectives/Plan to Provide Mentoring:

Strengths

- The candidate described clear short-term goals- learn qualitative methods for behavioral intervention design, use of social media platforms for promoting adolescent behavior change and clinical trials for testing behavioral interventions that map on well with the training plan and the proposed research.
- The training plan consists of coursework, didactic interactions with mentors, directed readings and on the implementation of the research study.
- The candidate proposed a Scientific Advisory Board comprised of 4 experienced clinicians and researchers who will meet yearly to help the mentoring team assess the candidate's progress
- Mentors clearly stated the regularity with which they will meet with the candidate, the format and duration of the meetings
- The proposed training in the responsible conduct of research and good clinical practice are appropriate.

Weaknesses

- The candidate's long-term career goal of becoming an expert in the adaptation and evaluation of interventions to improve transition care for adolescents living with HIV is somewhat narrow
- The components of the training, particularly the social media component is not adequately described.
- The proposed deliverables for each aim are publications. Publications are important, but measuring progress requires milestones and timelines. Without these milestones and timelines, progress is difficult to assess.
- There was some confusion regarding how attendance at the ATN meetings and participating in work group will result in training in the use and adaptation of social media for behavioral intervention. The applicant references the ATN's I-Tech study, Dr. Mayer, the candidate's link to the ATN, does not address the applicant's involvement with the ATN or the I-tech study in his letter.

3. Research Plan:

Strengths

- The proposed research plan maps well onto the candidates stated training goals.
- The study uses a phased approach that is rigorous, appropriate for the target population and grounded in theory.
- Each of the 3 aims is designed to help the candidate develop the skills that he identifies as needing in order to achieve research independence. Aim is designed to build skills on qualitative methods; Aim 2 on intervention development; and Aim 3 in clinical trials design and execution.
- The scientific premise of the proposed study is strong. The candidate clearly describes the scope of the problem, adequately justifies the approach (use of social media to reach the target population) and provides a strong rationale for conducting the study in South Africa
- The candidate has addressed biological variables. The proposed participants are adolescents living with HIV; both male ans females will be included.

Weaknesses

- There was insufficient description of the proposed in-depth qualitative guide. Sample content areas should have been provided
- The applicant raised the potential for cross contamination, but did not provide steps for minimizing this threat.

4. Mentor(s), Co Mentor(s), Consultant(s), Collaborator(s):

Strengths

- The candidate has proposed a strong mentoring team, that appears highly supportive of the candidate. The team is composed of 4 mentors, each with needed expertise in the candidate's stated training areas
- The majority of the mentors' letters are strong and clearly delineate their role in the K, their level of involvement, means and frequency of interaction.
- Dr. Haberer, the primary mentor, is an internationally known expert in technology-based assessments and intervention. She has been mentoring the candidate for a few years and appears highly committed to help advance him towards research independence.

Weaknesses

- Given the candidate's need for training in social media interventions and adaptation of social media to promote behavior change, the application would have been strengthened had the relevant mentors provided more detail in how this training would be provided and how progress would be measured.

5. Environment and Institutional Commitment to the Candidate:

Strengths

- The institutional commitment to the candidate is strong. The letter clearly states that the applicant will dedicate 75% effort to the K, that his position in the department is not contingent on receipt of this grant, and that the institution will work to ensure that the candidate has the mentoring, oversight and facilities needed to conduct the research.
- The remaining 25% of the candidate's effort will be divided into teaching, administration and clinical duties. All of which are appropriate to the advancement of the candidate's career.
- The research environment at Mass General and Harvard Medical School are outstanding. The candidate will have the necessary physical and computing facilities and the administrative and statistical support to complete the work.
- Given the diversity and talent of researchers at these institutions, the applicant will have access to numerous other collaborators who could help him to advance.
- The environments of the collaborating institutions in South Africa are also appropriate and will facilitate completion of the study. Furthermore, the applicant has had previous experience working at the hospital.

Weaknesses

- None noted

Protections for Human Subjects:

Acceptable Risks and Adequate Protections

Data and Safety Monitoring Plan (Applicable for Clinical Trials Only):

Acceptable

Inclusion of Women, Minorities and Children:

- Sex/Gender: Distribution justified scientifically
- Race/Ethnicity: Distribution justified scientifically
- For NIH-Defined Phase III trials, Plans for valid design and analysis: Not applicable
- Inclusion/Exclusion of Children under 18: Including ages < 18; justified scientifically

Vertebrate Animals:

Not Applicable (No Vertebrate Animals)

Biohazards:

Not Applicable (No Biohazards)

Training in the Responsible Conduct of Research:

Acceptable

Comments on Format (Required):

- varied format

Comments on Subject Matter (Required):

- appropriate subject areas

Comments on Faculty Participation (Required; not applicable for mid- and senior-career awards):

- appropriate faculty participation

Comments on Duration (Required):

- appropriate

Comments on Frequency (Required):

- appropriate

Budget and Period of Support:

Recommend as Requested

CRITIQUE 3

Candidate: 2
Career Development Plan/Career Goals /Plan to Provide Mentoring: 1
Research Plan: 3
Mentor(s), Co-Mentor(s), Consultant(s), Collaborator(s): 2
Environment Commitment to the Candidate: 1

Overall Impact: This is a resubmission of a K23 application proposing to develop and pilot a social media intervention to promote the transition to adult care and health outcomes among perinatally infected adolescents in South Africa. Overall, I would say this application is very responsive to the prior critiques. This project seems highly significant, given the large numbers of perinatally infected adolescents in South Africa, and documented challenges with their transition to adult HIV care and associated health outcomes. The candidate is highly accomplished with a proven track record of hands-on clinical experience with HIV in South Africa and a developing research program related to the continuum of care for adolescents living with HIV. He has already obtained intramural funding, a KL2, and an R21 to develop this program of research. Although he has now better justified additional training, there is still some question of whether he really requires another 4 years of career development support to continue on this trajectory. However, the training plan he proposes in this resubmission will give him important new skills that will no doubt enhance the work. He has assembled an excellent group of mentors and advisors to provide him with the needed training in qualitative research methods, social media intervention development, and clinical trial methodology. Overall the scope and the aims of the research—formative qualitative research, followed by intervention development research using an iterative approach, followed by a small pilot RCT--appear feasible and very appropriate for a K mechanism. There were a few weaknesses that slightly lowered my scores. The evidence presented that a social media intervention has the potential to increase social support

from peers and clinicians and thereby improve the transition to adult care seemed a bit weak. Differences between male and female adolescents seemed inadequately considered in the research plan. Given that the candidate has already been receiving mentorship from mentors listed on this application for a couple of years, one wonders how much support for this mentorship is really needed. Overall, however, I had high enthusiasm for this greatly strengthened application.

1. Candidate:

Strengths

- The candidate has very strong expertise in pediatric infectious disease, the adolescent continuum of care, and the South Africa context.
- The candidate has particularly strong hands-on experience with HIV and adolescents in South Africa, having established a teen clinic and provided clinical care for this population for five years.
- The candidate currently has a Harvard KL2 and an NICHD R21 regarding the adolescent transition to care in South Africa.

Weaknesses

- There is still some remaining doubt in my mind that this investigator requires 4 more years of career development support to be successful in his program of research.

2. Career Development Plan/Career Goals & Objectives/Plan to Provide Mentoring:

Strengths

- The training program on qualitative research methods, social media intervention development, and clinical trials of behavioral interventions is well thought out and appropriate to gain the needed skills.
- The training plan is now well linked to the research aims.
- The timeline is appreciated.

Weaknesses

- None noted.

3. Research Plan:

Strengths

- The significance of the project is high, given the large numbers of perinatally infected adolescents in South Africa, and documented challenges with their transition to adult HIV care and associated health outcomes. Only an estimated 10% are virally suppressed.
- If the project were to succeed in developing an acceptable and feasible intervention to assist these adolescents with successful transition to adult care, this would be an important advance for the field.
- The research plan is appropriate for the candidate’s career stage and is well matched with his training plan.
- The population, methods for developing the intervention, and the use of social media are novel.
- Interviewing adolescents who are lost to follow-up in Aim 1 seems important for gaining understanding and well thought out plans to trace and locate them are described.

Weaknesses

- I felt that the evidence presented from the literature was not as strong for a) lack of social support from peers and clinicians as a primary determinant of poor outcomes and b) social media interventions as effective methods for improving adolescent transition to care. This weakens the scientific premise a bit.
- Is social media use always associated with increased social support for adolescents?
- The proposed study uses several different frameworks/models to guide the work, which is sometimes confusing. Frameworks mentioned include SMART, UTAUT, and GotTransition's Six Core Elements of Healthcare Transition.
- Both male and female adolescents are included in the research plan. However, sex differences were not really addressed in the background or the analytical plans.

4. Mentor(s), Co Mentor(s), Consultant(s), Collaborator(s):

Strengths

- The candidate has an exceptionally strong team of mentors. Dr. Haberer (primary mentor) is an expert in ART adherence and mHealth technology research, key for the current project. She has a K24 for mentoring. Dr. Mayer (co-mentor) adds expertise in intervention design and implementation (among others). Dr. Psaros (co-mentor) will provide mentoring on qualitative research methods. Dr. Holden (co-mentor) is an expert in design and evaluation of health information technology.
- There is also an Advisory Board for the project, contributing other important areas of expertise. Dr. Archary is a pediatric infectious disease specialist focusing on adolescents based at the site in South Africa. Dr. Mellins is a clinical psychologist with behavioral research with adolescents and young adults, in particular, perinatally infected adolescents. Dr. Moosa is an adult infectious disease specialist in South Africa who deals with the challenges of adolescents transitioning to adult care. Dr. Parker is a biostatistician.

Weaknesses

- The candidate appears to already have been receiving mentorship from many of those on the mentorship and advisory team for some time (joint publications going back to 2013), raising the question of whether he really needs further mentorship from these persons.
- A large team of mentors/advisors has been assembled (although apparently 4 persons less than in the previous submission). It may be challenging to effectively obtain mentorship from all of them.
- Some of the mentor/advisor letters are a bit too generic and similar.

5. Environment and Institutional Commitment to the Candidate:

Strengths

- Scientific environments at Massachusetts General and University of KwaZulu-Natal are likely to be highly adequate for the project proposed.
- The Gandhi hospital appears to be an appropriate site for recruitment of this perinatally infected adolescent population.
- Massachusetts General and Harvard letters express strong commitment to the candidate.

Weaknesses

- The facilities and resources section was very brief and did not give much of a sense of the specific resources that will be available to support Dr. Zanoni.

Protections for Human Subjects:

Acceptable Risks and Adequate Protections

- Potential risks and strategies to mitigate each type of risk are well described.

Data and Safety Monitoring Plan (Applicable for Clinical Trials Only):

Acceptable

- Is it possible that an adolescent under 18 years of age may not have an adult caregiver? In that case can they provide informed consent for themselves?

Inclusion of Women, Minorities and Children:

- Sex/Gender: Distribution justified scientifically
- Race/Ethnicity: Distribution justified scientifically
- For NIH-Defined Phase III trials, Plans for valid design and analysis: Not applicable
- Inclusion/Exclusion of Children under 18: Including ages < 18; justified scientifically
- The sample includes both male and female adolescents aged 15-19 (changed from 13-24 in the prior version of the application.

Vertebrate Animals:

Not Applicable (No Vertebrate Animals)

Biohazards:

Not Applicable (No Biohazards)

Resubmission:

- This is a resubmission in which the investigator received multiple major critiques in several components of the application, and made major changes in response, including changing training goals, adding/cutting mentors, improving both the research and training plans, and reducing to a 4-year duration.
- I would say that the revised application is highly responsive to the critiques.

Training in the Responsible Conduct of Research:

Acceptable

Comments on Format (Required):

- Includes formal classroom courses, online courses, webinars, and in-person seminars.

Comments on Subject Matter (Required):

- The plan is comprehensive, including responsible conduct of research, good clinical practice, bioethics, and special webinars on IT safety, confidentiality, and security.

Comments on Faculty Participation (Required; not applicable for mid- and senior-career awards):

- Courses include faculty participation, although no mention is made of involvement of the mentors for this project in this aspect of the candidate's training.

Comments on Duration (Required):

- A total of 24-40 hours per year over the 4-year period seems more than adequate.

Comments on Frequency (Required):

- Each year of the award has multiple training activities, ranging from 24-40 hours per year.

Resource Sharing Plans:

Acceptable

Budget and Period of Support:

Recommended budget modifications or possible overlap identified:

- Budget for Dr. Zanoni to travel and spend time in South Africa seems a bit limited, with 2 trips in year 1, and only 1 trip per year in subsequent years of the project.

THE FOLLOWING SECTIONS WERE PREPARED BY THE SCIENTIFIC REVIEW OFFICER TO SUMMARIZE THE OUTCOME OF DISCUSSIONS OF THE REVIEW COMMITTEE, OR REVIEWERS' WRITTEN CRITIQUES, ON THE FOLLOWING ISSUES:

PROTECTION OF HUMAN SUBJECTS: ACCEPTABLE

INCLUSION OF WOMEN PLAN: (G1A) ACCEPTABLE

INCLUSION OF MINORITIES PLAN: (M5A) ACCEPTABLE

INCLUSION OF CHILDREN PLAN: (C1A) ACCEPTABLE

COMMITTEE BUDGET RECOMMENDATIONS: The budget was recommended as requested.

Footnotes for 1 K23 MH114771-01A1; PI Name: Zanoni, Brian C.

NIH has modified its policy regarding the receipt of resubmissions (amended applications). See Guide Notice NOT-OD-14-074 at http://grants.nih.gov/grants/guide/notice-files/NOT-OD-14-074.html. The impact/priority score is calculated after discussion of an application by averaging the overall scores (1-9) given by all voting reviewers on the committee and multiplying by 10. The criterion scores are submitted prior to the meeting by the individual reviewers assigned to an application, and are not discussed specifically at the review meeting or calculated into the overall impact score. Some applications also receive a percentile ranking. For details on the review process, see http://grants.nih.gov/grants/peer_review_process.htm#scoring.

MEETING ROSTER

The roster for this review meeting is displayed as an aggregated roster that includes reviewers from multiple CSR Special Emphasis Panels of the AIDS and Related Research Integrated Review Group for the 2018/01 council round.
This roster for CSR is available at:
http://public.era.nih.gov/pubroster/Reports?DOCTYPE=SEP&DESFORMAT=PDF&AGENDA_SEQ_NUM_P=334876
